# *UCP1* and *UCP3* Expression Is Associated with Lipid and Carbohydrate Oxidation and Body Composition

**DOI:** 10.1371/journal.pone.0150811

**Published:** 2016-03-09

**Authors:** Bruno A. P. Oliveira, Marcela A. S. Pinhel, Carolina F. Nicoletti, Cristiana C. Oliveira, Driele C. G. Quinhoneiro, Natália Y. Noronha, Júlio S. Marchini, Ana J. Marchry, Wilson S. Junior, Carla B. Nonino

**Affiliations:** 1 Department of Internal Medicine, Ribeirão Preto Medical School, University of São Paulo, Ribeirão Preto-SP, Brazil; 2 Department of Surgery and Anatomy, Ribeirão Preto Medical School, University of São Paulo, Ribeirão Preto-SP, Brazil; University of Santiago de Compostela School of Medicine - CIMUS, SPAIN

## Abstract

**Background/Objective:**

Uncoupling proteins (*UCPs*) are located in the inner membrane of mitochondria. These proteins participate in thermogenesis and energy expenditure. This study aimed to evaluate how *UCP1* and *UCP3* expression influences substrate oxidation and elicits possible changes in body composition in patients submitted to bariatric surgery.

**Subjects/Methods:**

This is a longitudinal study comprising 13 women with obesity grade III that underwent bariatric surgery and 10 healthy weight individuals (control group). Body composition was assessed by bioelectrical impedance. Carbohydrate and fat oxidation was determined by indirect calorimetry. Subcutaneous adipose tissue was collected for gene expression analysis. QPCR was used to evaluate *UCP1* and *UCP3* expression.

**Results:**

Obese patients and the control group differed significantly in terms of lipid and carbohydrate oxidation. Six months after bariatric surgery, the differences disappeared. Lipid oxidation correlated with the percentage of fat mass in the postoperative period. Multiple linear regression analysis showed that the *UCP1* and *UCP3* genes contributed to lipid and carbohydrate oxidation. Additionally, *UCP3* expression was associated with BMI, percentage of lean body mass, and percentage of mass in the postoperative period.

**Conclusions:**

*UCP1* and *UCP3* expression is associated with lipid and carbohydrate oxidation in patients submitted to bariatric surgery. In addition, *UCP3* participates in body composition modulation six months postoperatively.

## Introduction

Obesity is a public health concern in many parts of the world because it has been associated with increased risk of developing hypertension and diabetes[1]. The amount of macronutrients that is consumed and oxidized in the body determines energy storage. Establishing a negative energy balance is extremely important to stimulate weight loss in obese individuals[2].

Changes in lifestyle including diet modification and increased physical activity are strategies that aim at weight loss. However, these changes do not suffice in most cases, especially for individuals with severe obesity[3]. Bariatric surgery has been identified as the most useful tool to achieve weight loss and weight loss maintenance over time[4]. This procedure is recommended for individuals with body mass index (BMI) equal to or higher than 40 kg/m^2^ and with BMI higher than 35 kg/m^2^ when comorbidities exist[5].

Studies have suggested that genetic factors contribute to the development of obesity[6]. Indeed, approximately 40–70% of the variation in susceptibility to obesity can be attributed to genetics[7].

Uncoupling proteins (*UCPs*) are members of the protein family located in the inner membrane of mitochondria. These proteins participate in energy expenditure, thermogenesis, regulation of free fatty acids, and reduction of reactive oxygen species[8]. *UCP1* plays an important role not only in thermal regulation but also in energy balance and weight control[9]. A recent study conducted on experimental models of bariatric surgery detected the presence of increased body temperature and up-regulated brown adipose tissue *UCP1* protein expression levels after surgical intervention [10]. In addition to its action in the adipose tissue, *UCP1* contributes to the ability of the organism to oxidize substrates, metabolize lipids, and reduce weight, so high *UCP1* expression should prevent the development of obesity[11].

*UCP3* is also involved in energy metabolism regulation and weight control. Recent evidence has suggested that *UCP3* plays an important part in modulating the use of lipid and glucose as energy substrate[12, 13]. Gaining better understanding of the role *UCPs* play in controlling and maintaining energy substrate oxidation could help to manage obesity.

This study aimed to investigate how *UCP1* and *UCP3* expression influences substrate oxidation and body composition in patients submitted to bariatric surgery.

## Materials and Methods

### Ethics statement

This investigation was approved by the Research Ethics Committee of the Clinical Hospital of Ribeirão Preto Medical School, University of São Paulo, SP, Brazil (CAAE: 18973913.0.0000.5440). All the patients provided a written informed consent after receiving full explanation about the study.

### Study setting and patient selection criteria

This longitudinal study comprised 13 women with obesity grade III (BMI > 40 kg/m^2^) submitted to bariatric surgery by Roux-en-Y gastric bypass (RYGB) (Group 1-Bariatric Surgery) and 10 healthy weight individuals (BMI between 18.5 kg/m^2^ and 24.9 kg/m^2^) (Group 2-Control). This study did not include patients submitted to a modification of the standard surgical technique, patients who missed follow up with a multidisciplinary team, pregnant women, and patients with thyroid disease, cancer, or psychiatric disorders.

RYGB consisted in creating a small gastric portion (30 to 50 ml) and performing an anastomosis of the gastric stump to the jejunum (both remaining loops measured about 100 cm). All the surgeries were open, and the same team of surgeons operated on the participants. Anthropometric, body composition, and indirect calorimetry data as well as adipose tissue sample were collected during the preoperative period and six months after the surgery. Individuals belonging to Group 2 (Control) were evaluated just once.

### Anthropometry and body composition

Weight was measured with an electronic platform FilizolaTM scale with precision of 0.1 kg and maximum capacity of 300 kg. A vertical shaft with 0.5-cm graduation was used to measure the height. BMI was calculated based on the weight and height measurements. Abdominal circumference was measured with an inextensible tape at the largest circumference between the last rib and the iliac crest. A monofrequency Quantum BIA 450Q-RJL Systems analyzer was used to evaluate body composition. Resistance and reactance values were placed in particular equations validated for the obese population of this study[14].

### Substrate oxidation

Indirect calorimetry on the QUARK-RMR device (COSMED, Rome, Italy) helped to determine oxygen (O_2_) consumption and carbon dioxide (CO_2_) production during substrate oxidation. During the evaluation, the women lay awake in the supine position in a quiet room at a temperature between 21 and 24°C, under weak lighting. All the measurements were accomplished from 8:00 to 10:00 am. The women were advised to fast for six hours, not to do any physical exercise, and not to drink coffee or black tea 24 h before the assessment. The equipment was automatically calibrated with known gas concentrations before all the assessments, according to the manufacturer’s specifications.

Oxygen consumption (VO_2_) and carbon dioxide production (VCO_2_) were measured for 30 min. The measurements obtained in the first ten minutes were discarded, to ensure that the participant reached a steady state[15]. The Frayn equation (1983)[16] was used to calculate the carbohydrate and fat oxidation rates.

### Subcutaneous adipose tissue collection

During bariatric surgery, 2 g of abdominal subcutaneous adipose tissue was collected above the participants’ upper right umbilical scar for gene expression analysis. In the postoperative period, adipose tissue was collected by biopsy in the same region of the first procedure. For individuals belonging to Group 2, adipose tissue was collected during surgery of an umbilical hernia (incisional or epigastric) or gallstones without acute cholecystitis. The same medical team performed all the procedures.

### Gene expression

RNA was extracted from samples of subcutaneous adipose tissue by using the phenol-chloroform extraction method modified by Chomczynski & Sacchi (1987)[17]. The DNA complementary (cDNA) was synthesized in a 50-mL reaction vessel containing 100 ng of total RNA. A high-Capacity cDNA Reverse Transcription^®^ kit (Life Technologies) was employed according to the manufacturer's instructions.

Gene expression was analyzed in triplicate by qPCR conducted on the 7500 Fast Real PCR System (Applied Biosystems). Relative quantification of both *UCP1* and *UCP3*, toward the pooled sample, was calculated by using the comparative delta-delta-Ct method[18]. GAPDH and β-actin are the most stable reference genes for adipose tissue[19]. Therefore, during the assay, these compounds were used for normalization, to correct sample variations in RT-PCR efficiency and errors in quantification. The MIQE guidelines were followed[20].

### Statistical analysis

Descriptive statistics consisted of mean values and standard deviation. Data normality was verified by the Shapiro-Wilk test. Then, analysis of variance (ANOVA) with Tukey post hoc was performed. Pearson correlation between %fat mass and fat oxidation (g/day) was performed. Multiple linear regression was used to determine the contribution of genes to substrate oxidation and body composition. Statistical significance was set at p < 0.05. All the analyses were performed with the Statistical Package software for Social Sciences (SPSS version 20.0, Inc. Chicago. IL).

## Results

This study enrolled 13 obese women before and after six months of RYGB with a mean age of 32.7 ± 9.1 years (Group 1); it also included 10 healthy-weight women with a mean age of 34 ± 11 years (Group 2, control). The patients in Group 1 had percentage of excess weight loss equivalent to 22%. Table 1 lists the anthropometric and body composition variables and the volumes of oxygen and carbon dioxide. Considering the pre- and postoperative periods, participants in Group 1 presented significantly different anthropometric, body composition, and respiratory parameters.

**Table 1:** Anthropometry, body composition, and respiratory variables in grade III obese patients before and after (six months postoperatively) bariatric surgery (Group 1) and normal-weight individuals (Group 2).

	Group 1 Preoperatively (n = 13)	Group 1 Postoperatively (n = 13)	Group 2 (n = 10)
Weight (kg)	119.3 ± 15.0	92.5 ± 14.1^a^	56.7 ± 7.4^a^^,^^b^
Height (cm)	164.0 ± 7.4		161.2 ± 6.8
BMI (kg/m^2^)	44.5 ± 6.4	34.5 ± 5.7^a^	21.7 ± 2.1 ^a^^,^^b^
AC (cm)	125.5 ± 14.3	109.0 ± 14.4^a^	78.6 ± 8.7 ^a^^,^^b^
LBM (kg)	54.7 ± 4.3	50.0 ± 4.7^a^	39.5 ± 6.3 ^a^^,^^b^
%LBM	46.2 ± 3.3	54.6 ± 4.9 ^a^	69.4 ± 3.5 ^a^^,^^b^
FM (kg)	64.6 ± 11.4	42.5 ± 10.3^a^	17.4 ± 2.5 ^a^^,^^b^
%FM	53.8 ± 3.3	45.4 ± 4.9 ^a^	30.5 ± 3.5 ^a^^,^^b^
VO_2_ (L/min)	0.31 ± 0.04	0.27 ± 0.03	0.20 ± 0.04 ^a^^,^^b^
VCO_2_ (L/min)	0.21 ± 0.02	0.18 ± 0.02^a^	0.16 ± 0.02 ^a^^,^^b^
RQ	0.70 ± 0.05	0.69 ± 0.05	0.77 ± 0.09 ^a^^,^^b^

BMI: body mass index. AC: abdominal circumference. LBM: lean body mass. FM: fat mass. VO_2_: oxygen consumption. VCO_2_: carbon dioxide production. RQ: respiratory quotient.

^a^*p* < 0.05 as compared to Group 1 preoperatively.

^b^*p* < 0.05 as compared to Group 1 postoperatively.

**Fig 1:**
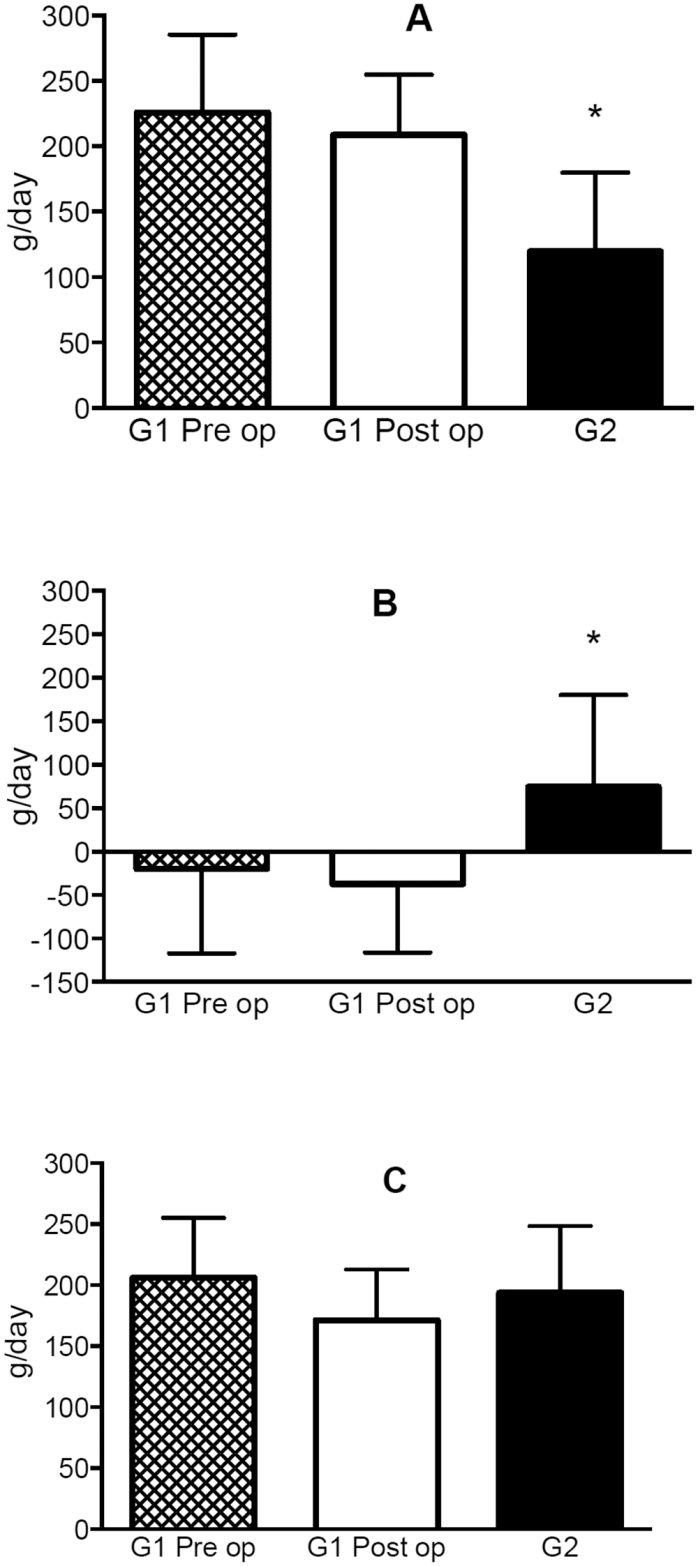
Substrate oxidation in patients submitted to bariatric surgery before and six months after the surgery and in the control group. A: lipid oxidation, B: carbohydrate oxidation, C: lipid + carbohydrate (lip + cho). Preop: preoperatively, Postop: postoperatively. *p < 0.05 Group 2 as compared to Group 1 Preop and Group 1 Postop.

There are significant differences between lipid and carbohydrate oxidation among patients who underwent bariatric surgery and control group. However no differences were observed when compared before and 6 months after bariatric surgery (Fig 1). Furthermore, the lipid + carbohydrate oxidation (lip+cho) presents no difference between the groups.

**Fig 2:**
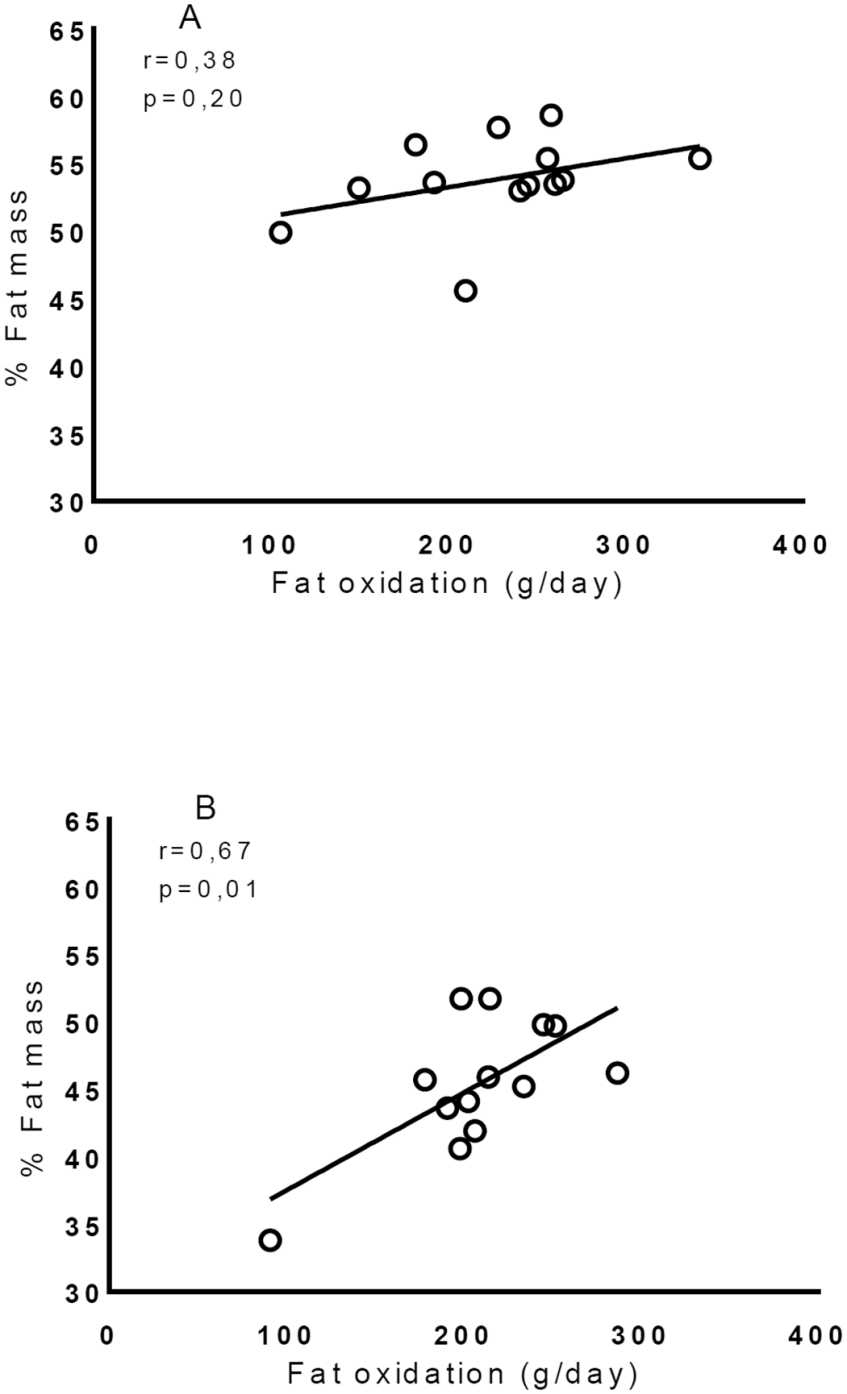
Correlation between lipid oxidation and percentage of fat mass. A: Preoperatively, B: Postoperatively.

The Fig 2 shows the correlations between lipid oxidation and percentage of fat mass (%FM) in patients submitted to RYGB. This figure evidences a positive relationship between the two variables in the postoperative period (r = 0.67; p = 0.01).

**Fig 3:**
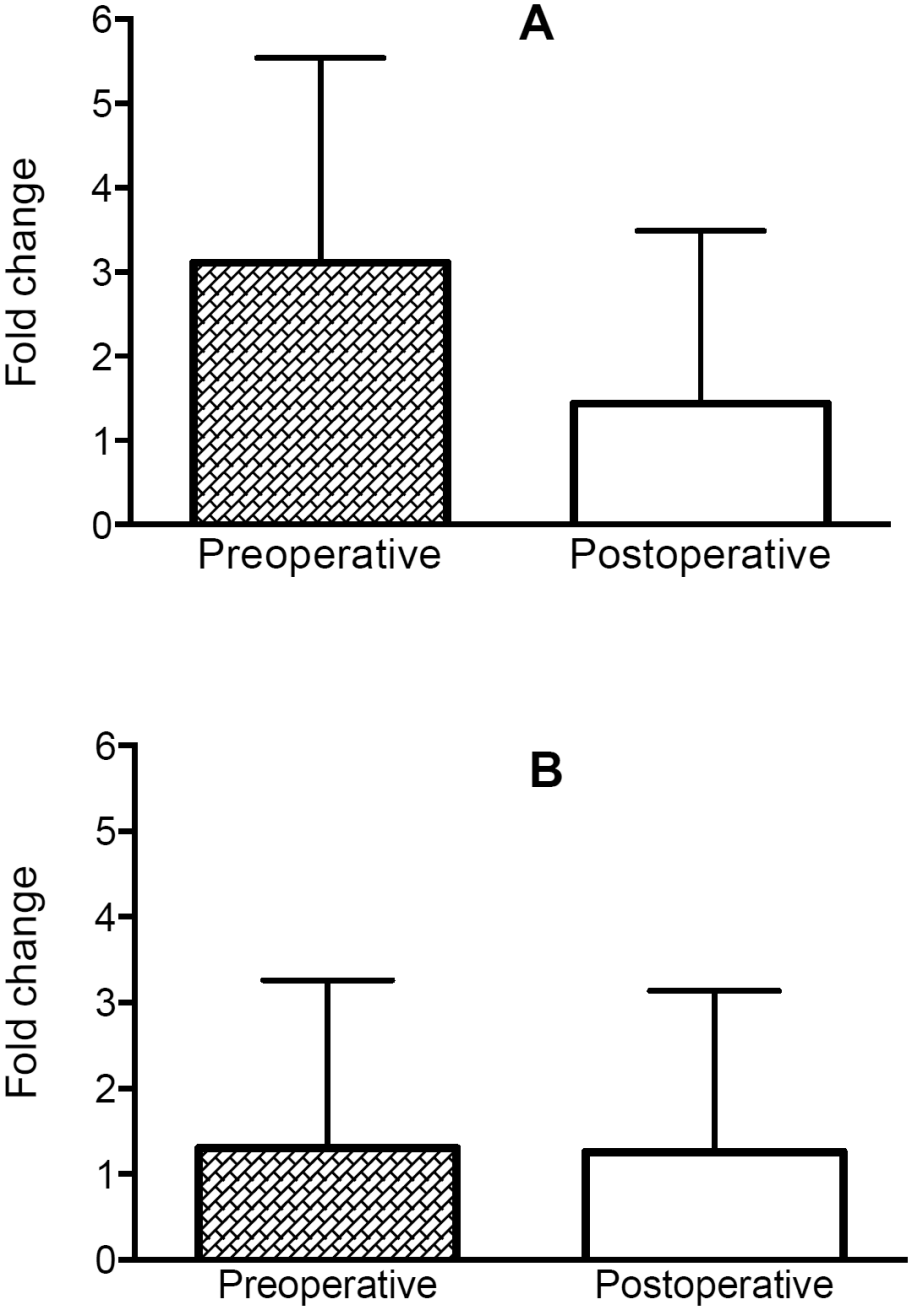
Relative gene expression in obese women preoperatively and six months after bariatric surgery. A: *UCP1*, B: *UCP3*.

The relative *UCP1* and *UCP3* gene expression was the same before and after bariatric surgery (Fig 3). However, multiple linear regression analysis revealed that *UCP1* and *UCP3* contributed to lipid and carbohydrate oxidation (Table 2).

**Table 2:** Contribution of *UCP1* and *UCP3* expression to lipid and carbohydrate oxidation.

	*UCP1*		*UCP3*	
Lipid oxidation	r^2^	*p*	r^2^	*P*
Preoperatively	0.430	**0.02**	0.288	**0.05**
Postoperatively	0.022	0.96	0.640	**0.01**
Carbohydrate oxidation				
Preoperatively	0.266	**0.04**	0.162	0.27
Postoperatively	0.076	0.82	0.767	**0.01**
Total oxidation				
Preoperatively	0.158	0.20	0.030	0.92
Postoperatively	0.133	0.70	0.529	**0.01**

regression adjusted by age. *p*<0.05.

Moreover, *UCP3* expression was associated with BMI, percentage of lean body mass (%LBM), and %FM in the postoperative period even after adjustment for age (Table 3).

**Table 3:** Contribution of *UCP3* to BMI, percentage of lean body mass (%LBM), and percentage of mass (%FM).

BMI	r^2^	*P*
Preoperatively	0.199	0.39
Postoperatively	0.486	**0.01**
% LBM		
Preoperatively	0.094	0.36
Postoperatively	0.496	**0.01**
% FM		
Preoperatively	0.094	0.36
Postoperatively	0.496	**0.01**

Regression adjusted by age. *p*<0.05.

## Discussion

This study showed that patients submitted to bariatric surgery presented different anthropometric, body composition, and respiratory variables before and six months after bariatric surgery. In contrast, the operated patients did not differ in terms of lipid and carbohydrate oxidation before and after RYGB. However, their preoperative lipid and carbohydrate oxidation parameters were different from these parameters in the control group. In addition, fat mass and lipid oxidation were positively associated in the postoperative period. Moreover, *UCP3* favorably impacted lipid and carbohydrate oxidation, BMI, %LBM, and %FM in the postoperative period.

Patients belonging to group 1 had a mean reduction of 16.5 cm in waist circumference six months after RYGB. Silva et al. (2013)[21] evaluated 98 obese subjects at the same postoperative period and found significantly reduced waist circumference (waist circumference decreased by 23.3 cm), which was similar to the findings reported by Carrasco et al. (2007)[22] in a sample of 31 patients (waist circumference decreased by 28.6 cm.)

RYGB provides an average loss of 35–40% of the baseline weight over a period of 12 to 24 months. The main purpose of this procedure is FM reduction, but LBM loss also takes place [23]. In the present study, LBM and FM decreased by 4.7 kg and 22.1 kg six months after surgery, respectively, which agreed with literature results [22–25].

Preoperative Group 1, postoperative Group 1, and Group 2 presented different respiratory variables (VO_2_ and VCO_2_). The respiratory quotient (RQ), which is the ratio between VO_2_ and VCO_2_, helped to identify which energy substrate the patient was oxidizing at the time of evaluation. RQ determination indicated that fat oxidation prevailed. Other studies have also shown lower RQ due to increased lipid oxidation after bariatric surgery[22, 26].

Here, preoperative Group 1 and the control group differed in terms of lipid and carbohydrate oxidation. These results contrasted with literature data. Labayen et al. (2004)[27] described different lipid oxidation in obese and healthy-weight individuals, whereas Nicoletti et al. (2013)[28] did not find any differences in carbohydrate oxidation. Studies showing the relationship between longitudinal changes in body composition and substrate utilization[29] pointed to a positive correlation between lipid oxidation and fat mass[27], which was similar to the findings of our study.

In the present study, *UCP1* and *UCP3* gene expression before and six months after RYGB was not different. Mingrone et al. (2003)[30] analyzed *UCP3* expression in the muscle tissue of 11 women before and 24 months after biliary-pancreatic derivation procedure and found reduced expression levels. On the other hand, Bracale et al. (2014)[31] did not verify any changes in *UCP3* expression in the skeletal muscle of women with severe obesity that were candidates for bariatric surgery.

In our study, *UCP1* and *UCP3* expression contributed to substrate oxidation. Analysis of the *UCPs* made it clear that intracellular energy from the fat cells played a significant role in energy metabolism, and that such proteins could be possible targets for personalized nutritional interventions and pharmacological approaches aiming at increasing fat oxidation and combating obesity[32, 33].

The *UCP3* gene associated with all the energy substrates in the postoperative period. Indeed, Cioff et al. (2009)[34] reported that *UCP3* has an important role in fatty acid oxidation and in preventing oxidative damage by mitochondrial reactive oxygen species (ROS). Dulloo and Samec (2001)[35] found that *UCP3* and lipid oxidation are markedly correlated. Our data resembled the results of Dulloo and Samec (2001)[35] and showed that the *UCP3* gene regulates lipid oxidation[35]. In this same context, *UCP1* expression in white adipose tissue evidenced that mitochondrial uncoupling can affect energy metabolism and glucose homeostasis[11]. Indeed, the present study showed that *UCP1* aids carbohydrate oxidation in the preoperative period.

The involvement of *UCP3* in protection from ROS-induced oxidative stress and in fatty acid oxidation rate enhancement suggests a protective role for this protein in obesity[36]. *UCP3* expression in the skeletal muscle, a tissue that represents 40% of the metabolic active mass and which contributes to energy homeostasis[36], strengthens this suggestion.

*UCPs* perform an important role not only in thermal regulation but also in the regulation of energy balance and body weight[9, 11]. However, few clinical studies have shown how *UCPs* influence body composition. The present study, which showed that *UCP3* expression is associated with BMI, %LBM, %FM in the postoperative period of bariatric surgery, represents an important contribution in this field.

In conclusion, *UCP1* and *UCP3* gene expression contributes to lipid and carbohydrate oxidation in patients submitted to bariatric surgery. In addition, the *UCP3* gene participates in body composition modulation six months after the surgical intervention.

## Supporting Information

S1 Table Status of anthropometry, body composition, oxidation of substrates, and gene expression. (XLS)
